# Sexual and reproductive health services utilisation amongst in-school young people with disabilities in Ghana

**DOI:** 10.4102/ajod.v10i0.671

**Published:** 2021-03-01

**Authors:** Akwasi Kumi-Kyereme

**Affiliations:** 1 Department of Population and Health, Faculty of Social Sciences, University of Cape Coast, Cape Coast, Ghana

**Keywords:** sexual and reproductive health services, young people, disabilities, Ghana, utilisation

## Abstract

**Background:**

Sexual and reproductive health (SRH) of young people including those with disabilities is a major public health concern globally. However, available evidence on their use of sexual and reproductive health services (SRHS) is inconsistent.

**Objective:**

This study investigated utilisation of SRHS amongst the in-school young people with disabilities (YPWDs) in Ghana using the healthcare utilisation model.

**Methods:**

Guided by the cross-sectional study design, a questionnaire was used to obtain data from 2114 blind and deaf pupils or students in the age group 10-24 years, sampled from 15 purposively selected special schools for the deaf and the blind in Ghana.

**Results:**

About seven out of every 10 respondents had ever utilised SRHS. The proportion was higher amongst the males (67.8%) compared with the females (62.8%). Young persons with disabilities in the coastal (OR = 0.03, 95% CI = 0.01–0.22) and middle (OR = 0.06, 95% CI = 0.01–0.44) zones were less likely to have ever utilised SRHS compared with those in the northern ecological zone. The blind pupils or students were more likely to have ever utilised SRHS than the deaf (OR = 1.45, 95% CI = 1.26–3.11).

**Conclusions:**

Generally, SRHS utilisation amongst the in-school YPWDs in Ghana is high but significantly associated with some predisposing, need and enabling or disabling factors. This underscores the need for policymakers to consider in-school YPWDs as a heterogeneous group in the design and implementation of SRHS programmes. The Ghana Education Service in collaboration with the Ghana Health Service should adopt appropriate pragmatic measures and targeted interventions in the special schools to address the SRH needs of the pupils or students.

## Introduction

Globally, sexual and reproductive health (SRH) of young people has been recognised as an important public health issue (Odo et al. 2018). Child marriage, unintended pregnancies and sexually transmitted infections (STIs), including human immunodeficiency virus (HIV), constitute an enormous burden on the health of young people (World Health Organization [WHO] 2018a). The evidence suggests that more than 1 million curable STIs are reported each day globally (WHO 2018b). According to global estimates of the WHO for 2016, there were approximately 376 million new infections of the four curable STIs – chlamydia, gonorrhoea, syphilis and trichomoniasis. From this figure, those aged 20–24 years recorded the highest proportion followed by those within the age group of 15–19 years (WHO 2018b). Trends of HIV suggest that one adolescent in the 15–19 years age group acquires HIV infection in every 2 minutes, and no decline in HIV-attributed death rates has been observed despite a reduction in the death rates in all population groups (Fatusi 2016; Shaw et al. 2016). In Ghana, the overall mean prevalence of HIV infection peaked at 3.6% in 2003 and declined to 1.6% in 2014. For those in the age group of 15–24 years, the mean prevalence was 1.8% in 2014, and 8.0% of female respondents and 9.0% of male respondents reported to have contracted STI in the 12 months before the 2014 Ghana Demographic and Health Survey (GDHS) (Ghana Statistical Service, Ghana Health Service, ICF Macro 2015)

Notwithstanding the enormous burden of SRH-related problems amongst young people (Shrivastava, Shrivastava & Ramasamy 2017), there have been inconsistent findings in their use of sexual and reproductive health services (SRHS). Generally, low utilisation of healthcare services amongst persons with disabilities (PWD) has been attributed to several impediments, including physical barriers, transport challenges, long waiting times, lack of confidentiality, need for an escort and disability-related stigma (Burke et al. 2017). Although some of the challenges are common amongst the various disability groups, some are also peculiar to specific types of disability. For example, blind persons are confronted with challenges, such as persons to aid them in the facilities and inaccessible healthcare facilities (Badu et al. 2018). With deaf persons, lack of privacy and confidentiality at SRH centres, lack of knowledge of healthcare providers on how to communicate and poor interpretation skills of sign language interpreters have been reported as barriers to using SRHS (Mprah 2013).

Globally, the SRH of young people has been given some attention (Aninanya et al. 2015). For instance, in 1994, at the International Conference on Population and Development (ICPD), social inclusion, human rights, and the needs and development of young people were brought to limelight (Jejeebhoy, Zavier & Santhya 2013). Subsequently, governments, especially those in the developing countries, have adopted various strategies to address the SRH needs of young people (Mbizvo & Zaidi 2010). The Government of Ghana passed the *Persons with Disability Act* 715 in 2006 and ratified the Convention on the Rights of PWD in 2012. These are geared towards improvement in the social, economic and political well-being of PWDs (Badu et al. 2019). However, according to Mprah, Anafi and Sekyere (2014), some of the SRH policies in Ghana do not pay any attention to the concerns of PWDs, and in a few cases where attention is given, it is either often cursory or focused on the negative.

Evidence suggests that studies have been conducted on various aspects of SRH amongst PWDs in Ghana over the last decade. Recently, some empirical studies have focused on access (including financial) (Badu et al. 2015, 2018), barriers to access (Badu, AgyeiBaffour & Opoku 2016a), challenges (Ganle et al. 2016), perspectives of PWDs on attitudes of health service providers (Badu, Opoku & Appiah 2016b), utilisation and satisfaction with health services (Abraham, Agyei-Baffour & Yarfi 2018) and other SRH-related issues (Badu et al. 2019; Karimu 2017).

A few of the recent studies focused on SRHS amongst adolescents and young people in special schools. For instance, Obasi et al. (2019) discussed SRHS amongst adolescents with disabilities, and Kumi-Kyereme, Seidu and Darteh (2020) assessed the challenges young people with disabilities (YPWDs) face in accessing SRHS. This article contributes to the discourse by investigating SRHS utilisation amongst the in-school YPWDs.

### Conceptual framework

**FIGURE 1:**
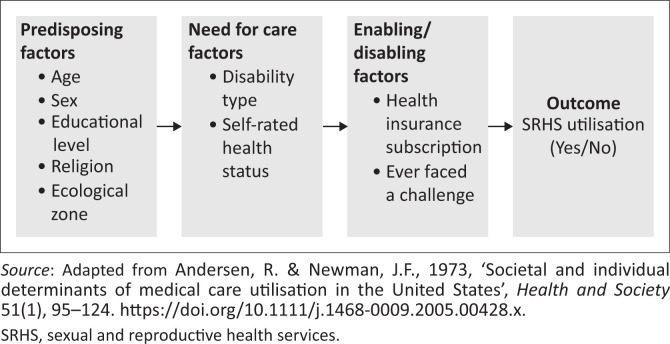
Conceptual framework.

The Healthcare Utilisation Model has been adapted as the conceptual framework for this study (see Figure 1). This model was propounded by Andersen and Newman (1973), but has subsequently been adapted (Andersen 1995). The model has been applied in various fields such as sociology, medicine, public health and psychology. There are three main components of the model: predisposing, need, and enabling or disabling factors. The model describes how these factors come to play to influence the utilisation of health services (Andersen 2008).

According to the model, predisposing factors are the demographic characteristics of individuals, such as age, sex, religion, education and ethnicity (Andersen 1995, 2008). The enabling or disabling factors are described as external to the individual, but they influence individuals’ decisions concerning the use of healthcare services. These factors include income or wealth, health insurance, travel time to health facilities, the means of transportation and waiting time (Babitsch, Gohl & Von Lengerke 2012). The need factors, according to the model, refer to perceptions of the seriousness of a disease or health condition (Andersen & Newman 1973; Babitsch et al. 2012). These include self-rated health, disability status, functional state and illness symptoms (Babitsch et al. 2012).

There is evidence that some scholars have critiqued the model. For instance, Wilson et al. (2005) were of the view that the model does not pay attention to cultural dimensions and social interactions. Another limitation of the model is its emphasis on healthcare utilisation or adopting health outcomes as a dichotomous factor, that is, present or not present. The overemphasis of need at the expense of health beliefs and social structure has also been criticised. However, from Andersen’s (2008) view, the model equally emphasises beliefs and social structure because need itself is a social construct. Despite these limitations, the model has been adapted to guide this study because of its strengths. For instance, the model considers healthcare utilisation from both the micro- (individual) and the macro-(community) level. This offers a better understanding of the demand and supply side opportunities and barriers rather than viewing health services utilisation as only a one-sided phenomenon.

In this article, SRHS utilisation is the dependent variable (Figure 1). The independent variables include the predisposing factors (sex, age, religion, educational level and ecological zone), the need factors (disability type and self-rated health status) and the enabling or disabling factors (health insurance subscription, ever faced a challenge). Factors, such as sex (Surood & Lai 2010), age, religion, ecological zone (Babitsch et al. 2012) and educational level (Dhingra et al. 2010), have proven to be significant in ascertaining the level of utilisation of healthcare services. Health insurance subscription has been shown, in other studies, to be a strong predictor of utilisation of healthcare services (Kyilleh, Tabong & Konlaan 2018).

## Methods

### Study setting and design

The data for this study were collected as part of a nationwide research project titled: *Sexual and Reproductive Health and Leisure Needs of YPWD in Ghana.* Using a cross-sectional study design, data were collected from the then 10 regions (now 16 regions) of Ghana between 01 November and 22 December 2017. Politically, Ghana is a unitary state divided into 16 administrative regions and 260 districts. The administrative regions are located in three broad ecological zones, namely, the Coastal, the Middle (forest belt) and the Northern (savannah). The results from the most recent Population and Housing Census conducted in Ghana show that the total population was 24 658 823 with a sex ratio of 95.2 men per 100 women. The population in the age group 10–24 years constituted 32.0% (7 849 520) of the total population. Persons with disabilities accounted for 3.0% (737 743) of the total population (350 096 men and 387 647 women) and 2.0% of the population were young people. The main religious groups in Ghana include Christians (67%), Muslims (17%) and Traditionalists (9%). About 53.0% of the population aged 15 years and older were literate in either English or a local language, and 34.0% of the population were literate in both (Ghana Statistical Service 2013).

### Target population and sampling procedures

There were 35 public and private special schools in Ghana when the data were collected in 2017. The special schools are classified as school for the deaf, school for the blind and school for the intellectually disabled. However, some of the schools are for both the blind and the deaf. This research study targeted only the schools for the blind and the deaf. All the 16 schools for the blind, and the deaf, comprising 14 schools for the deaf (including one Senior High School [SHS]) and two schools for the blind were purposively selected but the authorities in one of the schools declined to participate in the study.

This study targeted all the pupils and the students in the 15 special schools for the blind and the deaf who consented to participate in the study. The inclusion criteria for participation in the study included being a pupil or student, aged 10–24 years in the special schools for the blind and the deaf. However, pupils or students who had multiple disabilities, that is, both deaf and blind were excluded from the study. Those who were eligible in the sampled schools at the time of the data collection and consented to participate were included in the study.

There were a total of 4180 pupils or students in the 15 sampled special schools (Table 1). In each of the sampled schools, a screener was used to select pupils or students who met the eligibility criteria. Out of the total number of pupils or students screened, 2840 were eligible but 2114 (74.4%) of them consented to participate in the study. The number of male pupils or students who participated in the study was more (1163;55.0%) than the females (951;45.0%). The majority of the study participants (54.8%) were sampled from the special schools located in the Middle Ecological Zone.

**TABLE 1:** Distribution of study participants by sampled special schools.

No.	Name of school	Total enrolment	Number eligible	Number participated		
				Male	Female	Total
	**Coastal zone**					
1.	Cape deaf	364	296	112	81	193
2.	Salvation army	120	72	33	27	60
3.	State deaf	202	167	36	48	84
4.	Sekondi deaf	250	168	79	68	147
	*Sub-total*	936	703	260	224	484
	**Middle zone**					
5.	Secotech	250	250	73	69	142
6.	Demodeaf	439	221	102	83	185
7.	Akropong blind	367	217	83	86	169
8.	Kibi deaf	213	166	73	48	121
9.	Koforidua deaf	242	134	47	46	93
10.	Bechem deaf	331	233	111	74	185
11.	Ashanti deaf	552	366	141	122	263
	*Sub-total*	2394	1587	630	528	1158
	**Northern zone**					
12.	Wa deaf	200	140	80	54	134
13.	Wa blind	230	126	41	49	90
14.	Gbeogo	300	204	111	64	175
15.	Savelugu deaf	120	80	41	32	73
	*Sub-total*	850	550	273	199	472

	**Grand total**	**4180**	**2840**	**1163**	**951**	**2114**

### Method of data collection

The information present in the consent form was shared with all those who were eligible in a classroom. Pupils or students who were not willing to take part in the study were allowed to leave. Questionnaires (with a braille version for the blind) were administered to the blind and deaf pupils or students who had consented to participate in the study in different classrooms. In the schools with blind and deaf pupils or students, the questionnaires were administered to the blind and the deaf students in different classrooms. The participants were given time to respond to each question after it had been explained before the next question. The questionnaire that was adapted from an illustrative questionnaire for interview surveys was pre-tested in a special inclusive school in Cape Coast (www.who.int/reproductivehealth/topics/adolescence/questionnaire.pdf). The sections on the socio-demographic characteristics of the respondents and SRHS utilisation were used for this study.

Three field assistants were engaged and trained before the pre-testing of the questionnaire and the actual data collection exercise. The selection of the field assistants was based on their speciality in special education and their knowledge of SRH-related issues. One of them was a certified sign language interpreter, and two were Master of Philosophy students from the Departments of Population and Health and Special Education, University of Cape Coast.

### Data analysis

The administered questionnaires were checked for completeness and entered into Statistical Product and Service Solutions (SPSS, Chicago, IL, USA) version 23 software and subsequently exported to Stata (Stata Corporation, College Station, TX, USA) version 14.2 for analyses. The study employed both descriptive and inferential statistics in the analysis. Three sequential logistic regression models were constructed based on the categorisation of the independent variables into predisposing factors, need, and enabling or disabling factors after the descriptive analysis (see Figure 1). The first model analysed the association between the utilisation of SRHS and the predisposing factors (age, sex, educational level, religion and ecological zone). The second model assessed how the variables in Model I reacted with the introduction of the need for care factors (disability type and self-rated health status). In the third model, the enabling or disabling factors (health insurance subscription, ever faced a challenge) were added to the two models to make it complete. The results were presented as odds ratios (ORs), with their corresponding 95% confidence intervals (CI) signifying the level of precision. *P*-values less than 0.05 were considered to be statistically significant. The adapted conceptual framework for the study informed the choice of the inferential technique.

### Ethical considerations

The Institutional Review Board of the University of Cape Coast granted ethical clearance (UCCIRB/EXT/2017/13) for the study. Young people with disabilities who were eligible for the survey were provided with the informed consent form (with a braille version for the visually impaired), which had information on the purpose of the study, confidentiality, anonymity, the right to participate or decline to participate or withdraw from participating at any stage. They were encouraged to ask questions about the study and their participation. The heads of the sampled schools consented for those who were minors (10-17 years) before they assented. Written or verbal consent or assent was given by all those who took part in the study before they were enrolled.

## Results

### Socio-demographic characteristics of respondents

As shown in Table 2, the majority (61.7%) of the total respondents were in the age group 15-19 years (59.8% male and 63.9% female respondents). More than half (56.5%) of the respondents were from Junior High School (JHS) (55.2% male and 58.2% female respondents). All the respondents who were in SHS were deaf (6.3% male and 7.2% female respondents). The majority (81.3%) of the respondents include Christians (80.2% male and 82.7% female respondents) and 55.0% (54.3% male and 55.8% female respondents) resided in the Middle Ecological Zone of Ghana. The percentage distribution of the characteristics by type of disability does not vary much from that of the total respondents. For instance, the majority of the deaf and the blind male (59.8%) and female (63.9%) respondents were in the age group 15–19 years.

**TABLE 2:** Distribution of socio-demographic characteristics of respondents by sex and type of disability.

Variables	Sex and disability type						Total (*n* = 2114)
	Males			Females			
	Disability type			Disability type			
	Deaf (*n* = 1023)	Blind (*n* = 140)	Total (*n* = 1163)	Deaf (*n* = 804)	Blind (*n* = 147)	Total (*n* = 951)	
**Age (years)**							
10-14	18.9	20.7	19.1	20.9	21.1	20.9	19.9
15-19	60.7	53.6	59.8	64.1	63.3	63.9	61.7
20-24	20.4	25.7	21.1	15.0	15.6	15.2	18.4
**Educational level**							
Primary	38.2	40.7	38.5	35.3	30.6	34.6	36.8
JHS	54.6	59.3	55.2	56.1	69.4	58.2	56.5
SHS/technical	7.2	-	6.3	8.6	-	7.2	6.7
**Religion**							
No religion	2.4	5.0	2.8	1.7	1.4	1.7	2.3
Christianity	80.2	80.7	80.2	83.2	79.6	82.7	81.3
Islam	17.4	13.3	17.0	15.1	19.0	15.6	16.4
**Ecological zone**							
Northern	22.7	29.3	23.5	18.7	33.3	20.9	22.3
Middle	52.5	67.8	54.3	54.4	62.6	55.8	55.0
Coastal	24.8	2.9	22.2	26.9	4.1	23.3	23.7

*Source:* Field data, 2017

SHS, Senior High School; JHS, Junior High School.

### Utilisation of sexual and reproductive health services

Table 3 shows the proportions of the YPWDs, who self-reported to have ever utilised SRHS from a healthcare facility. The results reveal that about seven out of every 10 respondents had ever utilised SRHS. The proportion was higher amongst the male (67.8%) than amongst the female (62.8%) respondents. Also, the proportions varied by the type of disability and the socio-demographic characteristics. For instance, amongst the male respondents aged 15–19 years, 70.3% of the deaf compared with 61.8% of the blind students reported to have ever utilised SRHS. Similarly, amongst the female respondents who were located in the Middle Ecological Zone, 71.4% and 54.7% of the deaf and the blind students, respectively, had ever utilised SRHS.

**TABLE 3:** Utilisation of sexual and reproductive health services by disability type and socio-demographic characteristics of respondents.

Variables	Sex and disability type												Total	
	Males						Females						*n*	%
	Disability type						Disability type							
	Deaf		Blind		Total		Deaf		Blind		Total			
	*n*	%	*n*	%	*n*	%	*n*	%	*n*	%	*n*	%		
**All**	658	68.3	80	64.5	738	67.8	485	64.3	77	54.6	562	62.8	1300	65.6
**Age (years)**														
10-14	114	62.0	21	77.8	135	64.0	105	65.2	19	63.3	124	64.9	259	64.4
15-19	409	70.3	42	61.8	451	69.4	297	61.9	47	53.4	344	60.6	795	65.3
20-24	135	68.2	17	54.1	152	67.0	83	73.5	11	47.8	94	69.1	246	67.8
**Educational level**														
Primary	221	60.2	40	76.9	261	62.3	152	57.1	27	62.8	179	57.9	440	60.4
JHS	383	72.7	40	55.6	423	70.6	280	66.0	50	51.0	330	63.2	753	67.2
SHS/Technical	54	77.1	-	-	54	77.1	53	82.5	-	-	53	82.8	107	79.9
**Religion**														
No religion	15	65.2	4	66.7	19	65.5	9	69.2	1	50.0	10	66.7	29	65.9
Christianity	535	68.5	68	69.4	603	68.6	409	65.7	60	57.4	469	63.8	1072	66.4
Islam	108	67.5	8	40.0	116	64.4	67	56.8	16	59.3	83	57.2	199	61.2
**Ecological zone**														
Northern	140	65.4	16	41.0	156	64.4	79	54.1	26	53.1	105	58.9	261	53.9
Middle	361	71.2	62	76.5	423	71.9	294	71.4	47	54.7	341	68.5	764	70.4
Coastal	157	64.6	2	50.0	159	67.8	112	57.1	4	66.7	116	57.4	275	61.3

*Source:* Field data, 2017

SHS, Senior High School; JHS, Junior High School.

### Sexual and reproductive health services utilised

Out of the 1300 pupils or students who reported to have ever utilised SRHS, 1180 (90.8%) of them indicated the specific services accessed within the last 6 months preceding the survey (Table 4). The main SRHS received by both male and female respondents was the treatment for STIs (26.8%). However, amongst the male respondents the main SRHS received was contraceptives (42.7%) compared with STI treatment (29.0%) amongst the female respondents. The percentage distribution of the SRHS received by the deaf and the blind male pupils or students was about the same but varied amongst the female students. For instance, 31.3% of the blind female students received gynaecological services compared with 17.6% of their deaf counterparts. About 17.1% of both male and female students reported to have ever tested for HIV (31.9% male and 15.6% female students).

**TABLE 4:** Sexual and reproductive health services utilised by sex and disability type.

Variables	Sex and disability type						Total (*n* = 1180)
	Males			Females			
	Disability type			Disability type			
	Deaf (*n* = 617)	Blind (*n* = 74)	Total (*n* = 691)	Deaf (*n* = 422)	Blind (*n* = 67)	Total (*n* = 489)	
Contraceptives	33.6	37.8	42.7	14.2	9.0	13.5	17.0
STI treatment	24.6	31.1	25.4	27.7	37.3	29.0	26.8
Gynaecological services	-	-	-	15.4	31.3	17.6	15.8
Pregnancy test	-	-	-	14.9	7.4	13.9	13.1
Pregnancy termination	-	-	-	6.4	1.5	5.7	5.5
MCH	-	-	-	5.2	1.6	4.7	4.7
HIV testing	41.8	31.1	31.9	16.2	11.9	15.6	17.1

*Source:* Field data, 2017

MCH, maternal and child health; HIV, human immunodeficiency virus; STI, sexually transmitted infection.

### Multivariate analysis

Three sequential logistic regression models were built based on the conceptual framework employed for the study. As shown in Table 5, the pseudo *R*^2^ values for the three models were 0.02 (Model I), 0.04 (Model II) and 0.11 (Model III), suggesting that the models explain the variances in SRHS utilisation amongst YPWDs by 2.0%, 4.0% and 11.0%, respectively. Sex, educational level and ecological zone were significantly associated with the utilisation of SRHS in Model I. In Model II, ecological zone, disability type and self-rated health status were statistically significant. Ecological zone, disability type, self-rated health status, health insurance subscription and ever faced a challenge were also significantly associated with utilisation of SRHS in the complete Model (III) (Table 5).

**TABLE 5:** Logistic regression analysis of sexual and reproductive health services utilisation.

Variables	Model I		Model II		Model III	
	OR	CI	OR	CI	OR	CI
**Age (years)**						
10-14	Ref	-	Ref	-	Ref	-
15-19	0.85	0.65–1.10	1.05	0.68–1.62	0.97	0.49–1.89
20-24	0.76	0.53–1.08	1.35	0.72–2.54	1.37	0.55–3.37
**Sex**						
Males	Ref	-	Ref	-	Ref	-
Females	0.77**	0.64–0.93	0.79	0.57–1.10	1.08	0.66–1.75
**Educational level**						
Primary	Ref	-	Ref	-	Ref	-
JHS	1.48**	1.18–1.84	1.24	0.84–1.84	1.02	0.63–1.85
SHS/technical	2.53**	1.52–4.22	0.91	0.44–1.89	0.55	0.22–1.40
**Religion**						
No religion	Ref	-	Ref	-	Ref	-
Christianity	1.14	0.60–2.17	1.63	0.66–3.99	2.70	0.91–7.99
Islam	0.99	0.50–1.93	1.51	0.58–3.93	4.07	1.10–15.11
**Ecological zone**						
Northern	Ref	-	Ref	-	Ref	-
Middle	1.54***	1.21–1.96	0.71	0.43–1.18	0.07**	0.01–0.51
Coastal	1.13	0.85–1.49	0.40**	0.23–0.70	0.03***	0.01–0.25
**Disability type**						
Deaf	-	-	Ref	-	Ref	-
Blind	-	-	2.87*	1.28–6.44	1.94**	1.17–5.75
**Self-rated health status**						
Very good	-	-	Ref	-	Ref	-
Good	-	-	1.46*	1.02–2.09	1.69	0.99–2.87
Very bad	-	-	1.80	0.85–3.79	1.54**	1.12–3.97
Bad	-	-	1.06	0.56–2.01	0.75	0.31–1.79
**Health insurance subscription**						
Yes	-	-	-	-	Ref	-
No	-	-	-	-	0.63*	0.32–0.95
**Ever faced a challenge**						
Yes	-	-	-	-	Ref	-
No	-	-	-	-	0.58*	0.30–0.91
Pseudo *R*^2^	0.02	-	0.04	-	0.11	-

*Source:* Field data, 2017

Ref, reference category; OR, odds ratio; CI, confidence interval; SHS, Senior High School; JHS, Junior High School.

* , *p* < 0.10,

** ; *p* < 0.05;

*** , *p* < 0.001.

From Table 5, YPWDs in the Coastal (OR = 0.03, 95% CI = 0.01–0.25) and Middle (OR = 0.07, 95% CI = 0.01–0.51) Ecological Zones were less likely to have ever utilised SRHS compared with those in the Northern Ecological Zone. Blind pupils or students were about two times more likely to have ever utilised SRHS than deaf pupils or students (OR = 1.94, 95% CI = 1.17–5.75). Those who rated their health status as very bad were more likely to have ever utilised SRHS (OR = 1.54, 95% CI = 1.12–3.97) compared with those who rated their health status as very good. In addition, YPWDs who had not subscribed for health insurance recorded a lower probability of having ever utilised SRHS (OR = 0.63, 95% CI = 0.32–0.95) compared with those who had subscribed. Those who indicated that they never faced a challenge were less likely to use SRHS (OR = 0.58, 95% CI = 0.30–0.91) compared with those who ever faced a challenge (Table 5).

## Discussion

Whereas YPWDs have the same range of SRH needs and desires just like anyone else, they may encounter another layer of obstacle in assessing healthcare services as well as asserting their SRH rights because of the disabilities they have. This study sought to investigate SRHS utilisation amongst in-school young people who are blind and deaf in Ghana. The results show high levels of SRHS utilisation amongst the respondents studied. On the contrary, it was observed in a study conducted amongst PWDs in Accra that only one-fifth of them had utilised healthcare facilities (Abraham et al. 2018). However, the present findings must be viewed in the context of the unique positioning of the YPWDs in this study. They were all in-school young people and could be benefitting from school-based interventions and programmes on SRH (Jaleta, Assefa & Amentie 2017). Therefore, the high utilisation of SRHS could be because of a high level of awareness (Ayehu, Kassaw & Hailu 2016) and good knowledge (Obasi et al. 2019) about SRH.

Young people with disabilities in the Coastal and Middle Ecological Zones were less likely to use SRHS compared with those in the Northern Zone. There are existing north-south disparities in several development indicators in Ghana, which tend to favour the latter generally. Owing to this imbalance, several health interventions, including SRH, are ongoing in many communities in the Northern Zone. There is evidence that the government is collaborating with some NGOs to address the equity gaps in access to healthcare services, especially in the Northern Zone. Notable amongst the NGOs are Catholic Relief Services, West Africa AIDS Foundation and Alliance for Reproductive Health Rights (Hushie 2016). A recent review mapping of SRH education programmes in Ghana revealed that there were more of such interventions in the Northern Zone compared with the other parts of the country. Some of these interventions specifically targeted vulnerable young people, including those with disabilities (Amo-Adjei 2020). These contextual dynamics perhaps account for the spatial differences in better SRHS utilisation in the Northern compared with the Middle and Coastal Zones. The implication of this is that whilst affirmative action in SRH programming is important, there must be a deliberate effort not to leave anyone group behind in ways that become counterproductive.

The blind pupils or students were more likely to use SRHS compared with the deaf students. Even though persons with blindness may experience some physical accessibility challenges in seeking healthcare services, they are at an advantage in terms of effective communication with the providers. For the deaf, the space for communication is constricted, given the general lack of sign language experts in many healthcare facilities (Mprah 2013; Rugoho & Maphosa 2017). Amongst persons who reason that their needs may not be adequately served, they are not likely to utilise healthcare services and recommend the same to others in their networks or with people they share some major characteristics, even though geographical access may not be an obstacle. As asserted by Donabedian (1988), quality of service is an important trigger of re-visit, as well as referral to others. For programming, it is important that service providers are equipped with the skills to deal with all people, including those with one or another form of disability. Designating specific facilities to address the peculiar SRH needs of YPWDs may promote utilisation, especially amongst the deaf as shown in this study.

Self-rated health has been noted in the literature as a measure that predicts the utilisation of healthcare services (Tamayo-Fonseca et al. 2015). It was found that those who rated their health status as very bad were more likely to utilise SRHS compared with those who rated their health status as very good. Probably, the realisation of not being healthy served as a need factor for them to have their health screened, including SRH. In order to address the SRH needs of YPWDs comprehensively, especially those who might rate their health status as good based on their subjective assessment, preventive and promotive aspects of health should be emphasised in programming.

Young people with disabilities who had subscribed to health insurance recorded a higher probability of SRHS utilisation compared with those who had not subscribed. This finding confirms those of other previous studies, which revealed that ownership of health insurance affects utilisation of healthcare services (Boachie 2017; Kyilleh et al. 2018; Van der Wielen, Channon & Falkingham 2018). One of the key barriers to non-utilisation of healthcare services is the direct financial cost of healthcare (Dhillon et al. 2012). It is this quest to remove or minimise the effect of cost that many countries have diverse health insurance policies and programmes. Ghana introduced the National Health Insurance Scheme (NHIS) in 2003. Amongst the SRHS covered under the NHIS are counselling, testing of STIs and providing contraceptives. It is probably within this context that those insured used SRHS more frequently than the uninsured. Sexual and reproductive health services are critical for the well-being of YPWDs and for the additional vulnerabilities associated with disabilities. It is imperative that interventions, such as subscription waivers, are granted by the government to this population in order to enable them to access SRHS when needed.

Treatment of STIs was the main SRHS received by both the male and the female respondents, although the main SRHS received amongst the males was contraceptives. Probably, it is because YPWDs are susceptible and vulnerable to such infections (Suzanna et al. 2018). The fact that 13.2% of the young people reported ever testing for HIV might be a reflection of stigma associated with the virus in Ghana (Ogunbajo et al. 2018). That is, the fear of being stigmatised and victimised when their HIV status is known, young people may be discouraged from undergoing testing services. The extant literature recognises the misconceptions in many cultures, which tend to de-legitimise the sexuality of PWDs, especially in young people. They are sometimes not expected to express their sexuality, and therefore, those found to have negative SRH outcomes, such as STIs and unintended pregnancies, are stigmatised and censured (Manoj & Suja 2017).

### Strengths and limitations of the study

The survey, with a sample size of 2114, was conducted in selected special schools across the country. However, some limitations need to be acknowledged. Firstly, the study targeted in-school young people who are deaf or blind, and therefore, not representative of all YPWDs in the country. Secondly, the reporting of some behaviours could be biased in an attempt to provide culturally and socially desirable responses, despite the assurance of confidentiality and anonymity before the administration of the questionnaires. Thirdly, as discussed by Mprah (2013), there are various methodological concerns associated with the use of PWDs as study participants. For instance, some SRH-related concepts do not exist in the sign language (e.g. infection or contract and symptoms). These problems may have resulted in the mistranslation of some concepts and might have made the understanding of some of the survey items difficult for some participants. The research assistants minimised these possible challenges with translation by explaining the concepts with examples before the participants answered the questions. Also, there is the possibility that some of the transcriptions of the braille version might be inaccurate, although the transcribed data were double checked with the original answers to the transcribed ones in order to ensure accuracy. Despite these limitations, the findings of this study have policy implications on utilisation of SRHS amongst the in-school YPWDs in Ghana.

## Conclusions and policy implications

Generally, utilisation of SRHS amongst the in-school YPWDs in Ghana is high but significantly associated with some factors. These included predisposing (ecological zone), need for care (type of disability and self-rated health status) and enabling or disabling (health insurance subscription and ever faced a challenge) factors. The main SRHS received by YPWDs was STI treatment. For both the deaf and the blind male pupils or students, the main SRHS received was contraceptives compared with STI treatment amongst their female counterparts. The percentage distributions of the SRHS received by the deaf and the blind male students were about the same but varied amongst the female students.

The conclusion that SRHS utilisation amongst the in-school YPWDs is associated with some factors has policy implications. This underscores the need for policymakers to consider in-school YPWDs as a heterogeneous group in the design and implementation of SRH programmes. The range of SRHS received by the YPWDs suggests that they have SRH needs. The Ghana Education Service in collaboration with the Ghana Health Service should adopt appropriate pragmatic measures and targeted interventions in the special schools to address the SRH needs of all the pupils or students. These measures may include alerting pupils or students in the special schools about the range of SRHS available in healthcare facilities. Furthermore, healthcare providers could organise routine outreach SRHS for pupils or students in the special schools. For service providers, people who identify strongly with YPWDs may be drawn into frontline roles in the delivery of SRHS in the special schools.
